# Supplementary material for: Prevalence of skin Neglected Tropical Diseases and superficial fungal infections in two peri-urban schools and one rural community setting in Togo
Source: PLoS Negl Trop Dis. 2022 Dec 19;16(12):e0010697. doi: 10.1371/journal.pntd.0010697 (PMC9810153; doi:10.1371/journal.pntd.0010697)
Supplement: S1 Supplementary — (DOCX) [file pntd.0010697.s001.docx]

**Prévalence des maladies tropicales négligées à tropisme cutané et des mycoses cutanées superficielles dans deux écoles périurbaines et dans une communauté rurale au Togo**

Bayaki Saka^1^, Panawé Kassang^1^, Piham Gnossike^1^, Michael G Head^2^*, Abla Séfako Akakpo^1^, Julienne Noude Teclessou^1^, Yvette Moise Elegbede^3^, Abas Mouhari-Toure^4^, Garba Mahamadou^1^, Kokoé Tevi^1^, Kafouyema Katsou^1^, Koussake Kombaté^1^, Stephen L Walker^5^, Palokinam Pitché^1^

1. Service de dermatologie, CHU de Lomé (Togo)
2. Clinical Informatics Research Unit, Faculty of Medicine, University of Southampton, United Kingdom.
3. Service dermatologie, CHU de Kara (Togo)
4. Service de dermatologie, CHR de Kara (Togo)
5. Faculty of Infectious and Tropical Diseases, London School of Hygiene & Tropical Medicine, London, United Kingdom

*** Auteur correspondant:** Dr Michael Head, University of Southampton, [m.head@soton.ac.uk](mailto:m.head@soton.ac.uk)

**Résumé.**

**Introduction** : Les maladies tropicales négligées (MTN) à tropisme cutané sont endémiques et sous-diagnostiquées dans de nombreuses communautés à faibles revenus. L'objectif de cette étude était de déterminer la prévalence des MTN cutanées et des mycoses cutanées superficielles dans deux écoles primaires et dans une communauté rurale du Togo.

**Méthode** : Il s'agit d'une étude transversale qui s'est déroulée entre juin et octobre 2021. Les deux écoles primaires sont situées en périphérie de Lomé, la capitale. Le milieu communautaire était le village de Ndjéi, situé dans le nord-est du Togo. Les sites de notre étude ont été sélectionnés à dessein. Les participants de l’étude ont été examinés par des dermatologues. Le diagnostic des MTN cutanées était clinique.

**Résultats** : Au total, 1401 sujets ont été examinés dont 954 (68,1%) à Ndjéi, et 447 (31,9%) en milieu scolaire. Des infections cutanées ont été diagnostiquées chez 438 (31,3%) sujets, dont 355 (81%) en milieu communautaire. Parmi ces infections cutanées, 105 cas étaient des MTN cutanées (7,5%). Il s’agissait de 20 patients en milieu scolaire (4,5% des 447 participants) et 85 patients (8,9 % des 954 participants) en milieu communautaire. Tous milieux confondus, 68 cas de MTN cutanées ont été observées chez les 1020 (6,7%) enfants et 37 cas chez les 381 (9,7%) adultes. En outre, 333 cas de mycoses cutanées superficielles ont été observées (prévalence de 23,8 %). Les principales MTN cutanées diagnostiquées étaient la gale (n=86 ; 6,1%) et les cas suspects de pian (n=16, 1,1%). La prévalence de la gale était donc de 4,3% en milieu scolaire et de 7,0% en milieu communautaire. Un cas de lèpre a été diagnostiqué aussi bien en milieu scolaire qu’en milieu communautaire, et un cas suspect d'ulcère de Buruli en milieu communautaire. En milieu scolaire, cinq (6%) enfants atteints d'une MTN cutanées ont déclaré avoir été victimes de stigmatisation, parmi lesquels quatre avaient refusé d'aller à l'école. A Ndjéi, 44 (4,6%) patients ont déclaré avoir été stigmatisées et 41 (93,2%) d'entre eux ont manqué au moins un jour d'école ou de travail.

**Conclusion** : Cette étude montre que le fardeau de la gale et des infections cutanées telles que les mycoses superficielles est élevé en milieu scolaire et communautaire au Togo, y compris la stigmatisation. L'amélioration de l’accès aux soins et de l'éducation en milieux institutionnel et communautaire pourrait réduire la stigmatisation et encourager la déclaration précoce des cas d'infections cutanées à un centre de santé.

**Mots-clés** : Maladies tropicales négligées, MTN, infections cutanées, gale, Togo, lèpre, pian, infections fongiques, mycoses, ulcère de Buruli.

**Résumé de l'auteur**

Cet article porte sur un groupe d'affections appelées maladies tropicales négligées (MTN) à tropisme cutané. L'étude s’est déroulée au Togo, pays d’Afrique de l'Ouest, où il existe très peu de données sur les MTN. Des dermatologues ont procédé à des consultations dermatologiques d'élèves de deux écoles situées en périphérie de Lomé, la capitale, et des habitants d’une zone rurale du Togo, dans le nord-est du pays. Nous démontrons que la prévalence des mycoses cutanées superficielles est très élevée, et nous avons également diagnostiqué de nombreux cas de gale (l'une des MTN cutanées). Des cas de lèpre et d'ulcère de Buruli ont également été diagnostiqués. En outre, les niveaux de stigmatisation rapportés étaient élevés. Nos résultats montrent qu'il est essentiel de faire face à cette charge de morbidité pour améliorer la santé des populations, mais aussi pour réduire les conséquences socio-économiques de ces affections traitables.

**Introduction.**

Les maladies tropicales négligées (MTN) constituent un groupe de maladies qui sévissent dans de nombreux pays à revenu faible ou intermédiaire. Selon l'Organisation mondiale de la santé (OMS), plus d'un milliard de personnes, principalement dans les milieux à faible revenu, sont touchées par une ou plusieurs de ces maladies.^1^ Les enfants sont plus touchés que les adultes, et les facteurs de risque comprennent le bas niveau socio-économique, la promiscuité, la malnutrition, le climat et les précipitations.^2^ Les MTN cutanées peuvent entraîner une altération de la qualité de vie et affecter le bien-être psychologique en raison de l'apparence, de la déficience fonctionnelle, de la discrimination et de la stigmatisation des personnes atteintes.^3^ Les populations rurales sont à risque pour de nombreuses MTN cutanées, notamment la lèpre,^4^ l'ulcère de Buruli,^5^ le pian,^6^ et la gale.^7^ La gale, causée par l'acarien Sarcoptes scabiei, touche environ 455 millions de personnes dans le monde chaque année.^2^

Dans de nombreuses régions d'Afrique sub-saharienne où les MTN cutanées sont endémiques, il y a peu de dermatologues, ce qui réduit les perspectives de diagnostic efficace de ces maladies qui sont pour la plupart transmissibles.^8^ Les interventions éducatives sont connues pour faciliter la détection précoce de maladies stigmatisantes ou contagieuses telles que la lèpre, et pour prévenir leur propagation dans la communauté.^9^ Les études en milieu communautaire peuvent également aider au développement de nouvelles connaissances autour des approches intégrées de dépistage et de gestion de masse de certaines maladies endémiques.^10^

L'objectif de cette étude était de déterminer la prévalence des MTN cutanées dans deux écoles et dans un milieu communautaire rural, celui du village de Ndjéi, dans le nord-est rural du Togo. Ces résultats peuvent être utilisés pour fournir des données fiables susceptibles d'éclairer les décisions locales, nationales et internationales concernant la gestion des MTN cutanées.

**Méthodes**

Pour cette étude transversale, des cliniques mobiles ont été mises en place par des dermatologues au Togo, dans des sites scolaires et communautaires (Figure 1).

Les écoles primaires sont situées dans la région Maritime, dans les zones périurbaines à environ dix kilomètres au nord-ouest du centre de la capitale Lomé.

Le village de Ndjéi, qui compte environ 3 000 habitants, est situé dans le canton de Sirka, dans la région de Kara, à environ 400 km au nord de la capitale, Lomé. La grande ville la plus proche est Atakpamé, à environ 160 km. Les médicaments étaient offerts gratuitement aux participants (voir annexe 1). Au besoin, les patients étaient orientés vers le service approprié du système de santé. Par exemple, les patients atteints de la lèpre étaient orientés vers les centres de soins dédiés à la prise en charge de la lèpre à l'hôpital de district. Les dermatologues, hommes et femmes, étaient présents dans les écoles et les communautés.


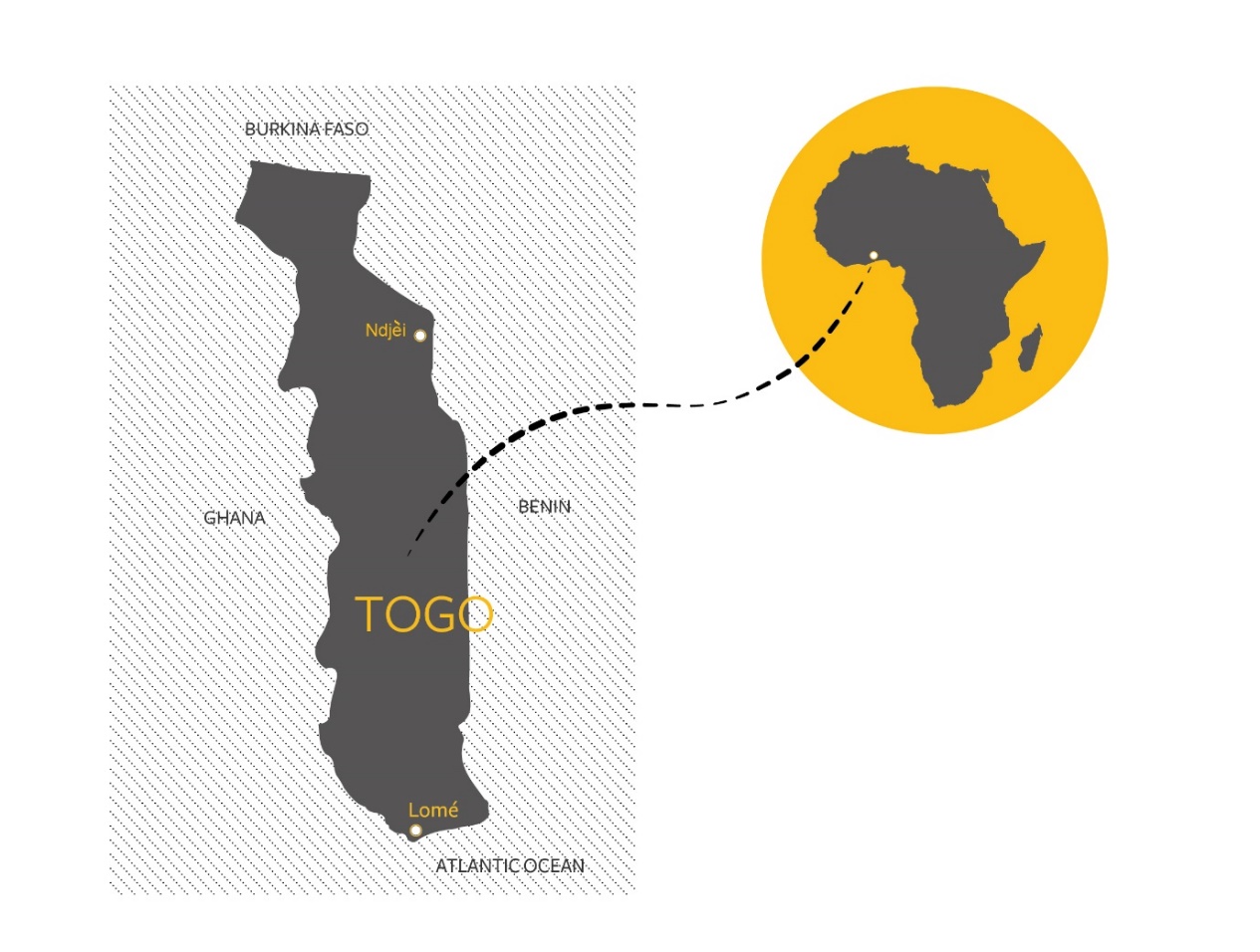


***Figure 1.*** *Situation de Lomé, capitale du Togo et lieu d'implantation des écoles, et de la communauté rurale de Ndjei.* *Image dessinée au sein de notre groupe de recherche, l'unité de recherche en informatique clinique de l'Université de Southampton*

**Milieu scolaire**

Les consultations se sont déroulées sur trois jours en juin 2021 dans les écoles primaires publiques d’Afiadegnigban et de Kpédevikopé. Les participants ont été examinés dans une salle privée par un dermatologue, avec un avis d’un deuxième dermatologue si nécessaire (par exemple pour confirmer un diagnostic). Chacun des cinq dermatologues avait au moins cinq ans d'expérience dans la pratique dermatologique. L'examen de la peau a duré environ dix minutes (avec les mesures de protection contre le Covid 19). L'équipe de dermatologues était assistée par deux infirmières et quatre agents de santé communautaires sur chaque site.

**Milieu communautaire**

Le directeur préfectoral de la santé a informé le personnel médical du centre de santé de Ndjéi (infirmiers, agents de santé communautaire) de la venue d'une équipe de dermatologues du 25 au 29 octobre pour des consultations dermatologiques. Le chef de village a ensuite informé la population. La population de Ndjéi est estimée à 3 000 personnes. L'équipe de l'étude a estimé qu'elle pourrait consulter environ un tiers de la population durant l'étude. L'activité principale de la population de Ndjéi est agricole. Un dermatologue s'est rendu à l'école primaire pour examiner systématiquement tous les enfants et leurs parents/tuteurs, et un autre au collège de Ndjéi. Le centre de santé du village a été utilisé comme lieu de consultation où deux dermatologues ont examiné toutes les personnes qui se présentaient pour une vaccination (programme national de vaccination, Covid-19) ou pour toute autre consultation (paludisme, gastro-entérite, etc). Par ailleurs, l'équipe a installé une clinique temporaire à 3 km du centre de santé, dans une église. Les ménages ont été invités pour un examen dermatologique. La sélection des ménages s'est faite à dessein, sur conseil des agents de santé locaux (par exemple, s'il fallait exclure un ménage). Sur les 25 ménages sélectionnés, 19 (76%) ont accepté de participer à l'étude. On s’assurait que les participants n'avaient pas été examinés sur d'autres sites. Les équipes ont respecté les mesures anti-COVID-19.

**Taille des échantillons**

Compte tenu d'un nombre d'élèves estimé à 392 et 390 dans chaque école, d'une marge d'erreur de 3%, d'un niveau de confiance de 95%, nous avons calculé une taille d'échantillon requise de 287 (marge d'erreur de 3%, niveau de confiance de 95%). Pour la communauté, compte tenu d'une population estimée à 3000 personnes, nous avons calculé une taille d'échantillon requise de 788 (marge d'erreur de 3%, niveau de confiance de 95%).

**Diagnostic des MTN cutanées**

Ont été considérées comme MTN cutanées : les dermatoses figurant sur la liste de l'OMS (https://www.who.int/neglected_diseases/skin-ntds/en/). Il s'agit de : Ulcère de Buruli ; leishmaniose cutanée ; leishmaniose cutanée post-kala-azar ; lèpre ; filariose lymphatique (lymphoedème et hydrocèle) ; mycétome ; onchocercose ; gale ; pian ; et mycoses sous-cutanées. L'équipe a également enregistré la présence de mycoses cutanées superficielles (par exemple, la teigne). Le diagnostic des MTN cutanées était exclusivement clinique, ainsi l'ulcère de Buruli et le pian étaient des cas suspects.

**Collecte et analyse des données**

Les données ont été recueillies à l'aide d'un formulaire standardisé pour chaque participant (annexe 2, 3). Les données recueillies comprenaient des informations sociodémographiques (âge, sexe, niveau d'éducation, nombre de personnes par foyer), des données cliniques (signes fonctionnels, type de lésions, siège des lésions), le diagnostic et des informations sur la stigmatisation. La stigmatisation était notée si la personne déclarait ressentir de la désapprobation ou des préjugés à cause de sa dermatose. La question figurant sur le formulaire de collecte des données était la suivante : "Si une infection cutanée est diagnostiquée, l’individu a-t-il ressenti une stigmatisation en raison de son infection ?" (Annexe 3). Le dermatologue demandait si la personne était traitée différemment ou injustement en raison de son infection cutanée, une approche courante pour obtenir une indication sur les niveaux de stigmatisation.^11^

Si un diagnostic de gale était posé, d'autres données étaient recueillies sur la présentation clinique et le siège des lésions. Les données ont été saisies dans le logiciel EPIDATA, version française 3.1. Des analyses descriptives ont été réalisées à l'aide de R© version 3.3.2 et les résultats ont été présentés sous forme de graphiques, tableaux, fréquences et pourcentages. Les variables quantitatives ont été décrites par des moyennes (± écart-type) et les variables qualitatives par des fréquences et des pourcentages.

**Déclaration d'éthique**

Cette étude a été approuvée par le Comité de bioéthique pour la recherche en santé au Togo (annexe 2, référence : 012/2021/CBRS du 5 mai 2021), ainsi que par le comité d'éthique de l'Université de Southampton (annexe 3, référence ERGO 63498). Le consentement écrit des adultes a été obtenu. Pour les mineurs, y compris les adolescents, le consentement a été signé par un parent ou un tuteur majeur après une explication dans une langue qu'ils comprenaient (français ou autre langue locale).

**Résultats**

Des examens dermatologiques ont été faits chez au total 1401 personnes dont 954 (68,1%) dans la communauté (Ndjéi). Dans cette communauté (Ndjéi), 296 (31%) participants ont été vus à l'école primaire, 220 (23,1%) au collège, 351 (36,8%) au centre de santé et 87 (9,1%) à la clinique temporaire. En milieu scolaire à Lomé, 447 enfants (31,9%) ont été examinés sur un total de 782 enfants inscrits (donc 57,2% de la population scolaire étaient ici participants).

La moyenne d’âge des sujets était de 10±2,6 ans (extrêmes : 5 et 17 ans) en milieu scolaire et de 19±15 ans (extrêmes : 1 et 90 ans) en milieu communautaire. Le sex-ratio (H/F) était de 0,9 dans les deux lieux. Des infections cutanées ont été diagnostiquées chez 438 (31,3 %) patients, dont 355 (81 %) en milieu communautaire (tableau 1).

**Tableau 1** : MTN cutanées et mycoses cutanées observées, N (%)

|  | **Milieu scolaire (n=447)** | **Milieu communautaire**  **(n=954)** | **Total**  **(N=1401)** |
| --- | --- | --- | --- |
|  |  | | |
| **MTN cutanée** |  |  |  |
| Oui | 20 (4,5) | 85 (8,9) | 105 (7,5) |
| Non | 427 (95,6) | 869 (91,1) | 1296 (92,5) |
| **Type de MTN cutanée** |  |  |  |
| Ulcère de Buruli | 0 (0) | 1 (0.1) | 1 (0,1) |
| Lèpre | 1 (0.2) | 1 (0,1) | 2 (0,1) |
| Scabiose | 19 (4,3) | 67 (7,0) | 86 (6,1) |
| Pian | 0 (0) | 16 (1,7) | 16 (1,1) |
| **Mycose cutanées observées** |  |  |  |
| Total des infections mycosiques | 63 (14,1) | 270 (28,3) | 333 (23,8) |
| *Teigne* | 16 (3,6) | 108 (11,3) | 124 (8,9) |
| *Pityriasis versicolor* | 44 (9,8) | 156 (16,4) | 200 (14,3) |
| *Dermatophytie cutanée* | 3 (0,7) | 6 (0,6) | 9 (0,6) |
| **Total des infections cutanées observées** | 83 (18,6) | 355 (37,2) | 438 (31,3) |
| **Participants sans infections cutanées** | 364 (81,4) | 599 (62,8) | 963 (68,7) |

On a observé 105 cas de MTN cutanées (7,5%) dont 20 cas dans les écoles (4,5% des 447 participants) et 85 (8,9%) à Ndjéi. Sur l'ensemble des milieux et selon l’âge, 68 cas de MTN cutanées (6,7%) ont été observées chez les enfants et 37 cas (9,7%) chez les adultes.

On a observé 333 cas de mycoses cutanées superficielles (23,8 % sur l'ensemble des sites dont 63 (14,1 %) dans les écoles et 270 cas (28,3 %) à Ndjéi. Sur l'ensemble des sites et en fonction de l'âge, on a recensé 259 cas de mycoses cutanées superficielles (25,4 %) chez les enfants et 74 (19,4 %) chez les adultes. Il y a eu deux cas de lèpre, tous deux multibacillaires (MB). Un cas suspect d'ulcère de Buruli a été diagnostiqué, à un stade évolué.

Parmi ces patients, 14 (3,2%) avaient au moins deux infections cutanées en même temps. La MTN cutanée la plus fréquente était la gale, observée chez 86 patients (6,1%) et le pian suspecté chez 16 patients (1,1%) (tableau 1). La prévalence de la gale était de 8,1% chez les adultes contre 5,4% chez les enfants. La prévalence du pian était similaire dans tous les groupes d'âge (1% chez les adultes contre 1,2% chez les enfants). Les mycoses cutanées superficielles étaient plus fréquentes chez les enfants (25,4% chez les enfants contre 19,4% chez les adultes) (tableau 2). En milieu scolaire, cinq (6%) enfants atteints de MTN cutanées ont déclaré être stigmatisés, et quatre d'entre eux avaient refusé d'aller à l'école pendant un ou plusieurs jours. A Ndjei, 44 (4,6%) patients ont déclaré avoir été stigmatisés et 41 (93,2%) d'entre eux ont manqué au moins un jour d'école ou de travail.

**Tableau 2** : MTN cutanées et mycoses cutanées observées, différenciées entre enfants et adultes, N (%)

|  | **Enfants (˂18 ans)**  **(n=1020)** | **Adultes (≥18 ans)**  **(n=381)** | **Total**  **(N=1401)** |
| --- | --- | --- | --- |
|  |  | | |
| **MTN cutanées** |  |  |  |
| Oui | 68 (6,7) | 37 (9,7) | 105 (7,5) |
| Non | 952 (93,3) | 344 (90,3) | 1296 (92,5) |
| **Type de MTN cutanée** |  |  |  |
| Ulcère de Buruli | 0 (0) | 1 (0,3) | 1 (0,1) |
| Lèpre | 1 (0,1) | 1 (0,3) | 2 (0,1) |
| Scabiose | 55 (5,4) | 31 (8,1) | 86 (6,1) |
| Pian | 12 (1,2) | 4 (1) | 16 (1,1) |
| **Mycose cutanées observées** |  |  |  |
| Total des infections mycosiques | 259 (25,4) | 74 (19,4) | 333 (23,8) |
| *Teigne* | 122 (11,9) | 2 (0,5) | 124 (8,9) |
| *Pityriasis versicolor* | 133 (13) | 67 (17.6) | 200 (14.3) |
| *Dermatophytie cutanée* | 4 (0,4) | 5 (1,3) | 9 (0,6) |
|  |  |  |  |
| **Total des infections cutanées observées** | 327 (32,1) | 111 (29,1) | 438 (31,3) |
| **Participants sans infections cutanées** | 693 (67,9) | 270 (70,9) | 963 (68,7) |

L'âge moyen des patients atteints de gale en milieu scolaire était de 10±2 ans (entre 5 et 13 ans) et de 20±14 ans (entre 1 et 72 ans) à Ndjei. Dans tous les cas de MTN cutanées, le prurit était le maître symptôme chez tous les patients. Il était à recrudescence nocturne chez 77 patients atteints de gale (89,5%), et 78 (90,7%) ont rapporté du prurit chez les membres du foyer. Les principales lésions cutanées étaient des papules (77 patients ; 89,5%), des lésions de grattage (74 patients ; 86,0%), des érosions/ulcérations (39 patients ; 45,3%) et des nodules scabieux (31 patients ; 36%) (tableau 3, annexe 4). Ces lésions étaient principalement situées sur les fesses (68 cas ; 79,1%), les poignets (65 ; 75,6%) et les espaces interdigitaux (56 ; 65,1%). Les lésions étaient impétiginisées chez 36 patients et eczématisées chez 12 autres. Trente-six patients (41,9%) ont déclaré être stigmatisés à cause de la gale et 11 d'entre eux (30,6%) ont rapportés avoir manqué au moins un jour d'école ou de travail.

**Tableau 3** : Lésions élémentaires et sites lésionnels chez les patients atteints de gale, N (%)

|  | **Milieu scolaire**  **(n=19)** | **Milieu communautaire**  **(n=67)** | **Total**  **(N=86)** |
| --- | --- | --- | --- |
|  |  | | |
| **Age (année)** | 10±2,6 | 19±14 | **-** |
| **Sexe** |  |  |  |
| Masculin | 10 (52,6) | 29 (43,3) | 39(45,3) |
| Féminin | 9 (47,4) | 38 (56,7) | 47 (56,3) |
| **Type de lésions** |  |  |  |
| Papules | 11 (57,9) | 66 (98,5) | 77 (89,5) |
| Lésions de grattage | 14 (73,7) | 60 (89,6) | 74 (86) |
| Erosions/Ulcérations | 13 (68,4) | 26 (38,8) | 39 (45,3) |
| Nodules scabieux | 10 (52,6) | 21 (31,3) | 31 (36) |
| Vésicules/bulles | 6 (32,6) | 11 (16,4) | 17 (19,8) |
| Pustules | 9 (47,4) | 3 (4,5) | 12 (14) |
| Sillons scabieux | 3 (15,8) | 2 (3) | 5 (5,8) |
| Autres lésions | 0 (0,0) | 3 (4,5) | 3 (3,5) |
| **Siège des lésions** |  |  |  |
| Fesses | 14 (73,7) | 54 (80,6) | 68 (79,1) |
| Poignets | 13 (68,4) | 52 (77,6) | 65 (75,6) |
| Espaces interdigitaux | 14 (73,7) | 42 (62,7) | 56 (65,1) |
| Seins | 6 (31,6) | 27 (40,3) | 33 (38,4) |
| Avant-bras | 5 (26,3) | 26 (38,8) | 31 (36) |
| Bras | 2 (10,5) | 24 (35,8) | 26 (30,2) |
| Cuisses | 0(0) | 21 (31,3) | 21 (24,4) |
| Organes génitaux externes | 4 (21,1) | 15 (22,4) | 19 (22,1) |
| Région péri-ombilicale | 2 (10,5) | 15 (22,4) | 17 (19,8) |
| Paume des mains | 0 (0) | 5 (7,5) | 5 (5,8) |
| Pieds | 0 (0) | 3(4,5) | 3 (3,5) |
| Autres | 4 (21,1) | 9 (13,4) | 13 (15,1) |
| **Complications** |  |  |  |
| Impétiginisation | 3 (15,8) | 33 (49,3) | 36 (41,9) |
| Eczématisation | 2 (10,5) | 10 (15) | 12 (14) |
| **Stigmatisation** |  |  |  |
| victimes de stigmatisation  Victimes de stigmatisation ayant manqués au moins un jour de travail ou d’école | 3 (15,8)  2 (66,7) | 33 (49,3)  9 (27,3) | 36 (41,9)  11 (30,6) |
|  |  |  |  |

**Discussion.**

Cette étude a permis de déterminer la prévalence des MTN cutanées et des mycoses cutanées superficielles en milieu scolaire et communautaire au Togo. La prévalence des mycoses cutanées superficielles est très élevée d’une part et d’autre part, la gale et le pian étaient les MTN cutanées les plus fréquentes.

La prévalence des MTN cutanées varie fortement selon le type de milieu (communauté ou institution) et selon le pays. Dans notre étude, la prévalence des MTN cutanées était de 7,5%, soit moins que les 17,2% d'une étude menée dans un hôpital éthiopien.^12^ De nombreuses autres études se sont concentrées sur une seule ou un petit nombre de MTN, alors que la plupart de ces maladies partageant les mêmes facteurs de risque, peuvent être observées simultanément chez le même patient et pourraient sans doute être étudiées ensemble de manière plus efficace.^1^ Dans notre étude, 3,2% des participants présentaient deux MTN cutanées en même temps. Une étude ivoirienne a également rapporté l'association fréquente chez des de patients de plusieurs affections cutanées, en particulier des infections fongiques, avec un cas de lèpre.^13^ Les recommandations de la feuille de route de l'OMS sur les MTN indiquent que ces affections devraient être considérées ensemble plutôt que séparément.^1^

Les mycoses cutanées superficielles (23,8%) étaient l'infection cutanée la plus observée dans notre étude, suivies de la gale (6,1%) et du pian (1,1%). La liste des MTN cutanées de l'OMS incluait auparavant les " infections fongiques ".^14^ Cependant, une définition plus pragmatique des MTN cutanées fait généralement référence aux mycoses des tissus profonds ou sous-cutanés, comme le mycétome, plutôt que d'inclure également les mycoses cutanées superficielles comme la teigne. Une étude menée en Éthiopie a révélé une prévalence d'infection fongique de 21,1 %^12^, un niveau similaire à nos résultats. Aussi, une étude menée au Bénin a rapporté une prévalence plus élevée de 49 % pour les mycoses cutanées^15^.

Le diagnostic clinique du pian a été posé chez 16 patients dans le village de Ndjei, alors qu'aucun cas n'a été trouvé dans les écoles du milieu péri-urbain de Lomé. Le diagnostic du pian dans cette étude était clinique, et il est possible que la prévalence soit surestimée en raison du manque de confirmation par des tests diagnostiques. La présentation clinique du pian primaire peut être similaire à celle de la leishmaniose cutanée ou d'une maladie mycobactérienne.^16^ Dans une étude similaire en Côte d'Ivoire, sur 15 cas de pian cliniquement suspectés, le diagnostic n'a été confirmé que chez 8 patients.^13^

La prévalence de la gale dans les écoles (4,3%) est similaire à celle observée dans les études chez les écoliers en Égypte, au Nigeria et en Turquie (4,4%, 4,8% et 2,16% respectivement).^17-19^ En revanche, une prévalence plus élevée de 39,4% et 17,2% a été observée chez les écoliers en Inde et au Cameroun respectivement.^20,21^ La prévalence communautaire de la gale dans cette étude est plus élevée que dans le cadre scolaire, mais similaire aux 6% rapportés dans une communauté rurale tanzanienne,^22^ et supérieure aux 2,8% rapportés dans une étude camerounaise.^23^ Lorsque la prévalence en milieu communautaire est élevée, cela augmente les risques d'épidémies de gale dans le cadre scolaire, par exemple une étude ghanéenne a mis en évidence les classes surpeuplées et le partage des matelas comme un facteur probable contribuant à une épidémie de 92 cas dans une école.^24^

Le prurit était le principal symptôme de la gale chez nos patients, comme cela a également été rapporté ailleurs.^24, 25^ Le prurit, en particulier le prurit nocturne et le prurit familial, sont généralement l'une des caractéristiques les plus évocatrices de la gale.^2^ Les fesses, les poignets et les espaces interdigitaux ont été observés ici comme les principaux sièges de lésions de gale. Ce sont des sièges classiques pour la gale, bien que les lésions puissent siéger n’importe quelle partie du corps. Dans certains cas, le diagnostic peut être difficile si les lésions sont cachées sous les vêtements, non signalée par le patient ou non vu par l’agent de santé. Une meilleure connaissance des sièges les plus fréquemment touchés peut permettre de mettre en place des méthodes de formation simples qui facilitent le diagnostic^26^.

La surinfection bactérienne (41,9% des cas) était la principale complication de la gale dans notre étude. L'utilisation fréquente des médicaments traditionnels pour traiter les dermatoses pourrait également favoriser cette surinfection ou l'eczématisation.^27^ Lorsque le prurit est difficile à dissimuler, cela peut être stigmatisant ou conduire à l'exclusion sociale. Dans une étude éthiopienne, près d'un tiers des patients atteints de gale ont fait l’objet d'une stigmatisation.^7^ Dans notre étude et pour l'ensemble des MTN, 49 personnes ont déclaré avoir été victimes de stigmatisation. Cette stigmatisation et le problème d'absentéisme qui y est associé devraient inciter les autorités sanitaires à rendre le traitement des MTN cutanées gratuit, y compris les frais de consultation, et à fournir des médicaments contre les MTN cutanées. Cela pourrait contribuer à persuader la population de consulter.

Les données probantes concernant le diagnostic, la prise en charge et l'identification des MTN cutanées sont variables, et les données de prévalence peuvent être peu concluantes ou obsolètes. Les études sur la gale se concentrent plus souvent sur les épidémies ou se déroulent en milieu institutionnel.^24, 28^ Cela est dû en partie au fait que des charges de morbidité plus élevées y ont été observées, mais aussi à leur relative simplicité par rapport aux grandes enquêtes communautaires. Une étude menée au Liberia a porté sur la gale dans la communauté et a rapporté une prévalence de 9,3 %,^29^ similaire à la prévalence communautaire de la gale de 7,2 % observée dans notre étude.

En 2022, l'OMS a révisé une feuille de route sur les MTN^1^, avec des objectifs de réduction nette et d'élimination de certaines MTN d'ici 2030, et une section consacrée à leur épidémiologie et à leur prévalence. Une grande partie de cette ambition mondiale dépend des dons de médicaments tels que l'ivermectine, et de leur intégration dans les administrations de médicaments de masse (AMM). Il est probable que les AMM auront plus d'impact dans les zones à forte endémie, et les données sur la prévalence communautaire sont donc vitales. Dans les régions où la charge de morbidité est plus faible, l'OMS indique que la surveillance et les soins en établissement peuvent être plus appropriés.^1^

Il existe peu de données sur la prévalence institutionnelle ou communautaire des MTN au Togo. Le pays a connu des succès importants avec les programmes de lutte, ayant éliminé la dracunculose (2011), la filariose lymphatique (2017) et la trypanosomiase humaine africaine (2020). Les données de cette étude montrent que les MTN cutanées telles que la gale et les mycoses cutanées superficielles sont hautement prévalentes et pourraient être plus difficiles à éliminer. La gale peut être contrôlée par l'administration massive d'ivermectine (qui est également utilisée pour lutter contre l'onchocercose). L'OMS élabore actuellement des directives provisoires concernant l'utilisation massive d'ivermectine pour lutter contre la gale.^30^

Les principales limites de notre étude sont inhérentes à la sélection de la population étudiée. Dans les écoles, certains parents/tuteurs étaient réticents à donner leur consentement pour la participation de leurs enfants aux consultations, ce qui explique pourquoi seulement 57,2 % des élèves y ont été examinés. Si les enfants souffrant d'une infection cutanée sont effectivement stigmatisés, il est possible qu'ils aient été plus susceptibles d'être absents au moment de notre étude, et donc que la prévalence soit sous-estimée. De même, dans la communauté rurale, certains participants assistaient à une campagne de vaccination au centre de santé. Ainsi, les caractéristiques des enfants absents de l'école et de ceux qui n'y sont pas allés à Ndjei peuvent biaiser les résultats. Par ailleurs, l'échantillon peut ne pas être représentatif de ces populations. Dans le cadre des programmes de santé scolaire et communautaire, tels que l'administration massive de médicaments ou le déploiement de vaccins, le taux de participation est généralement plus élevé là où la communauté s'est largement engagée.^31^ Un programme soutenu de promotion de la santé, dans le cadre scolaire et communautaire, peut encourager une approche proactive du comportement de recherche de soins, ainsi que la participation à des études de recherche. Les diagnostics étaient cliniques, et aucun autre examen paraclinique n'a été réalisé. Les diagnostics de pian et d'ulcère de Buruli sont donc suspectés plutôt que confirmés.

**Conclusion.**

Notre étude montre que le fardeau des MTN cutanées et des infections fongiques est élevé en milieu scolaire et communautaire au Togo. La gale était l'infection la plus fréquente. Une approche de gestion intégrée ainsi que des programmes d'administration massive de médicaments peuvent être efficaces pour contrôler ces MTN. Cependant, il faut également envisager des approches visant à réduire la stigmatisation associée, avec un meilleur accès aux soins et une éducation dans les établissements et les communautés qui encouragent la déclaration précoce des maladies à un centre de santé.

**Conflit d’intérêts**

Les auteurs ont déclaré qu'il n'y a pas de conflits d'intérêts.

**Remerciements**

Nous témoignons notre reconnaissance à l'Université de Southampton (Royaume-Uni) qui a financé ce projet de consultation communautaire et institutionnel. Nous remercions également les autorités administratives et sanitaires des districts sanitaires du Golfe et de la Binah qui ont autorisé la réalisation de cette étude. Nous remercions enfin Ashley Heinson, de l'Université de Southampton, pour ses efforts dans la cartographie des lésions de gale chez les patients. Nous tenons à rendre hommage au Dr Mwelecele Ntuli Malecela, directeur du département de lutte contre les MTN de l'OMS, décédé en 2022; qu'elle repose en paix.

**Contributions des auteurs**

BS a rédigé le projet de manuscrit, et a également effectué des consultations dermatologiques. MGH a conçu l'étude, rédigé le protocole de l'étude et les documents à soumettre aux comités éthiques, et révisé les versions initiales du manuscrit. PK a co-écrit le projet de manuscrit et a effectué les consultations dermatologiques. PG, ASA, JNT, YME, AMT, KT, KA ont effectué les consultations dermatologiques. GM, KK, PP ont soutenu la conception de l'étude, la rédaction du protocole et du manuscrit. SW a révisé le protocole, les documents à soumettre au comité éthique, le manuscrit et a apporté son expertise tout au long de l'étude. Tous les auteurs ont fait des commentaires sur le projet de manuscrit et ont approuvé la version finale.

**Disponibilité des données**

Les données peuvent être téléchargées sur https://doi.org/10.6084/m9.figshare.21276693.v1.

**References**

1 World Health Organisation. Ending the Neglect to Attain the Sustainable Development Goals: A road map for neglected tropical diseases 2021–2030. Geneva, 2020 https://www.who.int/neglected_diseases/Ending-the-neglect-to-attain-the-SDGs--NTD-Roadmap.pdf (accessed July 28, 2021).

2 Engelman D, Cantey PT, Marks M, *et al.* The public health control of scabies: priorities for research and action. *Lancet (London, England)* 2019; **394**: 81–92.

3 Weiss MG. Stigma and the Social Burden of Neglected Tropical Diseases. *PLoS Negl Trop Dis* 2008; **2**: e237.

4 Suzuki K, Akama T, Kawashima A, Yoshihara A, Yotsu RR, Ishii N. Current status of leprosy: epidemiology, basic science and clinical perspectives. *J Dermatol* 2012; **39**: 121–9.

5 Yotsu RR, Murase C, Sugawara M, *et al.* Revisiting Buruli ulcer. *J Dermatol* 2015; **42**: 1033–41.

6 Kazadi WM, Asiedu KB, Agana N, Mitjà O. Epidemiology of yaws: an update. *Clin Epidemiol* 2014; **6**: 119–28.

7 Dagne H, Dessie A, Destaw B, Yallew WW, Gizaw Z. Prevalence and associated factors of scabies among schoolchildren in Dabat district, northwest Ethiopia, 2018. *Environ Health Prev Med* 2019; **24**: 1–8.

8 Mitjà O, Marks M, Bertran L, *et al.* Integrated Control and Management of Neglected Tropical Skin Diseases. *PLoS Negl Trop Dis* 2017; **11**: e0005136.

9 Barreto JG, Guimarães L de S, Frade MAC, Rosa PS, Salgado CG. High rates of undiagnosed leprosy and subclinical infection amongst school children in the Amazon Region. *Mem Inst Oswaldo Cruz* 2012; **107 Suppl 1**: 60–7.

10 Engelman D, Fuller LC, Solomon AW, *et al.* Opportunities for Integrated Control of Neglected Tropical Diseases That Affect the Skin. *Trends Parasitol* 2016; **32**: 843–54.

11 Luck-Sikorski C, Roßmann P, Topp J, Augustin M, Sommer R, Weinberger NA. Assessment of stigma related to visible skin diseases: a systematic review and evaluation of patient-reported outcome measures. *J Eur Acad Dermatology Venereol* 2022; **36**: 499–525.

12 Abdela SG, Diro E, Zewdu FT, *et al.* Looking for NTDs in the skin; an entry door for offering patient centered holistic care. *J Infect Dev Ctries* 2020; **14**: 16S-21S.

13 Yotsu RR, Kouadio K, Vagamon B, *et al.* Skin disease prevalence study in schoolchildren in rural Côte d’Ivoire: Implications for integration of neglected skin diseases (skin NTDs). *PLoS Negl Trop Dis* 2018; **12**: e0006489.

14 World Health Organization. Archived webpage World Health Organization NTDs. 2020. https://web.archive.org/web/20200714092426/https://www.who.int/neglected_diseases/skin-ntds/en/ (accessed July 21, 2022).

15 Barogui YT, Diez G, Anagonou E, *et al.* Integrated approach in the control and management of skin neglected tropical diseases in Lalo, Benin. *PLoS Negl Trop Dis* 2018; **12**: e0006584.

16 Mitjà O, Hays R, Lelngei F, *et al.* Challenges in Recognition and Diagnosis of Yaws in Children in Papua New Guinea. *Am J Trop Med Hyg* 2011; **85**: 113.

17 Inanir I, Turhan Şahin M, Gündüz K, Dinç G, Türel A, Serap Öztürkcan D. Prevalence of skin conditions in primary school children in Turkey: differences based on socioeconomic factors. *Pediatr Dermatol* 2002; **19**: 307–11.

18 Salah Hegab D, Mahfouz Kato A, Ali Kabbash I, Maged Dabish G. Scabies among primary schoolchildren in Egypt: sociomedical environmental study in Kafr El-Sheikh administrative area. *Clin Cosmet Investig Dermatol* 2015; **8**: 105.

19 Ogunbiyi AO, Owoaje E, Ndahi A. Prevalence of skin disorders in school children in Ibadan, Nigeria. *Pediatr Dermatol* 2005; **22**: 6–10.

20 Sarkar M. Personal hygiene among primary school children living in a slum of Kolkata, India. *J Prev Med Hyg* 2013; **54**: 153–8.

21 Kouotou EA, Nansseu JRN, Kouawa MK, Zoung-Kanyi Bissek AC. Prevalence and drivers of human scabies among children and adolescents living and studying in Cameroonian boarding schools. *Parasites and Vectors* 2016; **9**: 1–6.

22 Henderson CA. Skin disease in rural Tanzania. *Int J Dermatol* 1996; **35**: 640–2.

23 Bissek ACZ, Tabah EN, Kouotou E, *et al.* The spectrum of skin diseases in a rural setting in Cameroon (sub-Saharan Africa). *BMC Dermatol* 2012; **12**. DOI:10.1186/1471-5945-12-7.

24 Kaburi BB, Ameme DK, Adu-Asumah G, *et al.* Outbreak of scabies among preschool children, Accra, Ghana, 2017. *BMC Public Health* 2019; **19**: 1–9.

25 Sanei-Dehkordi A, Soleimani-Ahmadi M, Zare M, Jaberhashemi SA. Risk factors associated with scabies infestation among primary schoolchildren in a low socio-economic area in southeast of Iran. *BMC Pediatr* 2021; **21**. DOI:10.1186/S12887-021-02721-0.

26 Osti MH, Sokana | Oliver, Lake S, *et al.* The body distribution of scabies skin lesions. *JEADV Clin Pract* 2022; **1**: 111–21.

27 Kobangué L, Guéréndo P, Abéyé J, Namdito P, Mballa MD, Gresenguet G. [Scabies: epidemiological, clinical and therapeutic features in Bangui]. *Bull Soc Pathol Exot* 2014; **107**: 10–4.

28 Cassell JA, Middleton J, Nalabanda A, *et al.* Scabies outbreaks in ten care homes for elderly people: a prospective study of clinical features, epidemiology, and treatment outcomes. *Lancet Infect Dis* 2018; **18**: 894–902.

29 Collinson S, Timothy J, Zayzay SK, *et al.* The prevalence of scabies in Monrovia, Liberia: A population-based survey. *PLoS Negl Trop Dis* 2020; **14**: e0008943–e0008943.

30 World Health Organization. Informal consultation on a framework for scabies control. 2019 https://www.who.int/publications/i/item/9789240008069 (accessed July 21, 2022).

31 Manyeh A, Ibisomi L, R R, Baiden F, Chirwa T. Exploring factors affecting quality implementation of lymphatic filariasis mass drug administration in Bole and Central Gonja Districts in Northern Ghana. *PLoS Negl Trop Dis* 2020; **15**: e0009341.

**Informations complémentaires**

Annexe 1. Liste des médicaments disponibles pendant l'étude

Annexe 2. Formulaire de collecte de données utilisé dans cette étude, version française.

Annexe 3. Formulaire de collecte de données utilisé dans cette étude, version anglaise.

Annexe 4. Données complémentaires et cartographie des sites des lésions de gale.
