# Supplementary material for: Prevalence of skin Neglected Tropical Diseases and superficial fungal infections in two peri-urban schools and one rural community setting in Togo
Source: PLoS Negl Trop Dis. 2022 Dec 19;16(12):e0010697. doi: 10.1371/journal.pntd.0010697 (PMC9810153; doi:10.1371/journal.pntd.0010697)
Supplement: S2 Supplementary — (DOCX) [file pntd.0010697.s002.docx]

| **Médicaments  Medicines** | **Quantité (boites) Quantity** |
| --- | --- |
| **Acaril-bial solution** | 150 |
| **Micozal crème** | 70 |
| **Forcan 150 MG gellule** | 150 |
| **Griséofulvine cp 500 MG (B/100)** | 14 |
| **Till solution** | 55 |
| **Azithrin gellule 250 MG** | 54 |
| **Prurex crème** | 50 |
| **Diprosone pommade** | 150 |
| **Alerid cp 10 MG** | 100 |
| **Vaseline salycilée 10%** | 50 |
| **Vaseline salycilée 15%** | 50 |

**PROPOSITION DE LISTE DE MEDICAMENTS A ACHETER POUR LES CONSULTATIONS DERMATOLOGIQUES EN MILIEU COMMUNAUTAIRE**
