# Supplementary material for: Prevalence of skin Neglected Tropical Diseases and superficial fungal infections in two peri-urban schools and one rural community setting in Togo
Source: PLoS Negl Trop Dis. 2022 Dec 19;16(12):e0010697. doi: 10.1371/journal.pntd.0010697 (PMC9810153; doi:10.1371/journal.pntd.0010697)
Supplement: S3 Supplementary — (DOCX) [file pntd.0010697.s003.docx]

**Prévalence des maladies cutanées tropicales négligées en milieu institutionnel et communautaire au Togo**

| Nom de la communauté / institution: …………………………………………………………….  Numéro d'identification du participant à l'étude:  Date de consultation / \| __ \| __ \| / \| __ \| __ \|  Identification du code: ___________________________________________  Membre de l'équipe de recherche remplissant ce formulaire: __________________  **________________________** | | | | | |
| --- | --- | --- | --- | --- | --- |
| **Section I: Données socio-démographiques** | | | | | |
| **QUESTIONS** | | **TERMES** | | **CODES** | **INSTRUCTIONS POUR REMPLIR** |
| I.1- Quel âge avez-vous? | | \|__\|__\| Ans  \|__\|__\| mois (s'il s'agit d'un nourrisson) | |  | Entrée de l'âge révolu |
| I.2 Combien de membres dans le ménage? | | _____ | |  | Entre nombre |
| I.3- Sexe | | Masculin | | 1 | Encerclez le code correspondant |
|  |  | Femme | | 2 |  |
| I.4- Profession | | Lycée | | 1 |  |
|  |  | Collège | | 2 |  |
|  |  | Primaire | | 3 |  |
|  |  | Ecole coranique | | 4 |  |
|  |  | Non scolarisé | | 5 |  |
|  |  | Autres (adultes) ...... ....... | | 6 |  |
|  | | | | | |
| Section II - Aspects cliniques de la maladie | | | | | |
| **QUESTIONS** | | **TERMES** | | **CODES** | **INSTRUCTIONS POUR REMPLIR** |
| II.1- Histoire connue du VIH? | Oui  Non | | 1  0 | |  |
| II.2 Présence de prurit? | Oui  Non | | 1  0 | |  |
|  | **Dermatoses infectieuses** | |  | |  |
| II.3 Diagnostic de toute maladie tropicale cutanée négligée | Ulcère de Buruli | |  | |  |
|  | Leishmaniose cutanée | |  | |  |
|  | Leishmaniose cutanée post-kala-azar | |  | |  |
|  | Lèpre | |  | |  |
|  | Filariose lymphatique | |  | |  |
|  | Mycétome | |  | |  |
|  | Onchocercose | |  | |  |
|  | Gale | |  | | Ajouter une note supplémentaire si incrustée / gale norvégienne |
|  | Pian | |  | |  |
|  | Maladie fongique | |  | |  |
| II.4 Avec une MTN diagnostiquée, une infection bactérienne secondaire a-t-elle été observée? | Oui  Non | | 1  0 | |  |
| II.5 Une infection bactérienne primaire distincte a-t-elle été diagnostiquée? | Oui  Non | | 1  0 | |  |
| II.6- Signes fonctionnels | | Prurit du corps entier | | 1 | Encerclez le code correspondant |
|  |  | Prurit du visage | | 2 |  |
|  |  | Prurit du cuir chevelu | | 3 |  |
|  |  | Prurit d'une autre partie du corps? (à préciser) | | 4 |  |
|  |  | Prurit familial / conjugal? | | 5 |  |
|  |  | Prurit nocture? | | 6 |  |
|  |  | Autres | |  |  |
| II.7- Quelles sont les lésions élémentaires trouvées chez le patient | | Papules | | 1 | Encerclez le code correspondant |
|  |  | Pustules | | 2 |  |
|  |  | Erosions/ulcérations | | 3 |  |
|  |  | Vésicules/bulles | | 4 |  |
|  |  | Lésions de grattage | | 5 |  |
|  |  | Nodules scabieux | | 6 |  |
|  |  | Sillons scabieux | | 7 |  |
|  |  | Autres......... | | 8 |  |
| II.8- Quels sont les sièges des lésions | | Espaces interdigitaux | | 1 | Encerclez le code correspondant |
|  |  | Poignets | | 2 |  |
|  |  | Cuisses | | 3 |  |
|  |  | Jambes | | 4 |  |
|  |  | Seins | | 5 |  |
|  |  | Fesses | | 6 |  |
|  |  | Organes génitaux externes | | 7 |  |
|  |  | Paumes des mains | | 8 |  |
|  |  | Plantes des pieds | | 9 |  |
|  |  | Bras | | 10 |  |
|  |  | Avant-bras | | 11 |  |
|  |  | Manches antérieures | | 12 |  |
|  |  | Région péri-ombilicale | | 13 |  |
|  |  | Autres | | 14 |  |
| II.9- Y a-t-il des signes associés à la gale (infection bactérienne secondaire; eczéma)? | | Eczéma: lésions érythémateuses et lésions squameuses (eczéma) | | 1 |  |
|  |  | Eczéma: lésions vésiculeuses | | 2 |  |
|  |  | Eczéma: lésions squameuses | | 3 |  |
|  |  | infection bactérienne secondaire: pustules | | 4 |  |
|  |  | infection bactérienne secondaire: suintement | | 5 |  |
|  |  | infection bactérienne secondaire: croûtes jaunâtres | | 6 |  |
|  |  | Autres | | 7 |  |
| II.10- Du prurit ou des signes d'infection parmi les membres du ménage? | | Oui  Non | | 1  0 | Encerclez le code correspondant |
| II.11 Si une infection cutanée est diagnostiquée, le participant a-t-il subi une stigmatisation en rapport avec son infection? | | Oui  Non  Infection cutanée non diagnostiquée | | 1  0  2 |  |
| II.12 Si «oui» à la question sur la stigmatisation, le participant n’a-t-il pas manqué à un ou plusieurs jours de travail ou d’école en raison de cette stigmatisation? | | Oui  Non | | 1  0 |  |
