# Supplementary material for: Prevalence of skin Neglected Tropical Diseases and superficial fungal infections in two peri-urban schools and one rural community setting in Togo
Source: PLoS Negl Trop Dis. 2022 Dec 19;16(12):e0010697. doi: 10.1371/journal.pntd.0010697 (PMC9810153; doi:10.1371/journal.pntd.0010697)
Supplement: S4 Supplementary — (DOCX) [file pntd.0010697.s004.docx]

**Prevalence of neglected tropical skin diseases in institutional and community settings in Togo**

| Name of the community / institution:: …………………………………………………………….  Identification number of the study participant::  Date of consultation / \| __ \| __ \| / \| __ \| __ \|  Code identification numbers: ___________________________________________  Member of the research team filling out this form: __________________  **________________________** | | | | | | | |
| --- | --- | --- | --- | --- | --- | --- | --- |
| **Section I: Demographics** | | | | | | | |
| **QUESTIONS** | | **TERMS** | | | | **CODES** | **INSTRUCTIONS FOR COMPLETING** |
| I.1- Age? | | \|__\|__\| Years  \|__\|__\| months (if individual is an infant) | | | |  | Enter the persons age |
| I.2 How many household members? | | _____ | | | |  | Entre the number |
| I.3- Sex | | Male | | | | 1 | Circle the correct code |
|  |  | Female | | | | 2 |  |
| I.4- Profession/student status | | High school | | | | 1 |  |
|  |  | Middle school | | | | 2 |  |
|  |  | Primary | | | | 3 |  |
|  |  | Koranic school | | | | 4 |  |
|  |  | Not in school | | | | 5 |  |
|  |  | Others (adult) ...... ....... | | | | 6 |  |
| For adults in community setting, type of profession | | | Unemployed/at home | | 1 | |  |
|  | | | Employed | | 2 | |  |
| For employed, add in type of employment | | | ____________________ | | 1 | | Free text field for later coding |
|  | | |  | |  | |  |
|  | | | | | | | |
| Section II - Clinical aspects of the disease | | | | | | | |
| **QUESTIONS** | | **TERMS** | | | | **CODES** | **INSTRUCTIONS FOR COMPLETION** |
| II.1- History of HIV? | Yes  No | | | 1  0 | | |  |
| II.2 Prescence of pruritus (itch)? | Yes  No | | | 1  0 | | |  |
|  | **Skin infections** | | |  | | |  |
| II.3 Diagnosis of any skin Neglected Tropical Diseases | Buruli ulcer | | |  | | |  |
|  | Cutaneous leishmaniasis | | |  | | |  |
|  | Post-kala azar cutaneous leishmaniasis | | |  | | |  |
|  | Leprosy | | |  | | |  |
|  | Lymphatic filariasis | | |  | | |  |
|  | Mycetoma | | |  | | |  |
|  | Onchocerciasis | | |  | | |  |
|  | Scabies | | |  | | | Ajouter une note supplémentaire si incrustée / gale norvégienne |
|  | Yaws | | |  | | |  |
|  | Other fungal disease | | |  | | |  |
| II.4 If an NTD is diagnosed, has a secondary bacterial infection been observed? | Yes  No | | | 1  0 | | |  |
| II.5 Has a distinct primary bacterial infection been diagnosed? | Yes  No | | | 1  0 | | |  |
| II.6- Functional signs | | Whole body itching | | | | 1 | Circle the correct code |
|  |  | Facial itching | | | | 2 |  |
|  |  | Itching of scalp | | | | 3 |  |
|  |  | Itching of another part of the body (specify) | | | | 4 |  |
|  |  | Family/household itching also ? | | | | 5 |  |
|  |  | Itching at night | | | | 6 |  |
|  |  | Other | | | |  |  |
| II.7- What are the types of lesions found in the patient? | | Papules | | | | 1 | Circle the correct code |
|  |  | Pustules | | | | 2 |  |
|  |  | Erosions/ulcerations | | | | 3 |  |
|  |  | Vesicles/bubbles | | | | 4 |  |
|  |  | Scratching lesions | | | | 5 |  |
|  |  | Scabies nodules | | | | 6 |  |
|  |  | Scabies burrows | | | | 7 |  |
|  |  | Others......... | | | | 8 |  |
| II.8- Where are the sites of any lesions? | | Between the digits/fingers | | | | 1 | Circle the correct code |
|  |  | Wrists | | | | 2 |  |
|  |  | Thighs | | | | 3 |  |
|  |  | Legs | | | | 4 |  |
|  |  | Breasts | | | | 5 |  |
|  |  | Buttocks | | | | 6 |  |
|  |  | External genitalia | | | | 7 |  |
|  |  | Palms of hands | | | | 8 |  |
|  |  | Torso | | | | 9 |  |
|  |  | Arms | | | | 10 |  |
|  |  | Forearms | | | | 11 |  |
|  |  | Upper arm | | | | 12 |  |
|  |  | Per umbilical region | | | | 13 |  |
|  |  | Other | | | | 14 |  |
| II.9- Are there any signs associated with scabies (secondary bacterial infection; eczema)? | | Eczema: erythematous lesions and scaly lesions (eczema) | | | | 1 |  |
|  |  | Eczema: vesicular lesions | | | | 2 |  |
|  |  | Eczema: scaly lesions | | | | 3 |  |
|  |  | secondary bacterial infection: pustules | | | | 4 |  |
|  |  | secondary bacterial infection: oozing | | | | 5 |  |
|  |  | secondary bacterial infection: yellow scabs | | | | 6 |  |
|  |  | Other | | | | 7 |  |
| II.10- Itching or signs of infection among household members? | | Yes  No | | | | 1  0 | Circle the correct code |
| II.11 If a skin infection is diagnosed, did the study participant experience stigma as a result of their infection? | | Yes  No  Skin infection not diagnosed | | | | 1  0  2 |  |
| II.12 If "yes" to the stigma question, did the study participant not attend one or more work or school days because of the stigma? | | Yes  No | | | | 1  0 |  |
