# Supplementary material for: Prevalence of skin Neglected Tropical Diseases and superficial fungal infections in two peri-urban schools and one rural community setting in Togo
Source: PLoS Negl Trop Dis. 2022 Dec 19;16(12):e0010697. doi: 10.1371/journal.pntd.0010697 (PMC9810153; doi:10.1371/journal.pntd.0010697)
Supplement: S5 Supplementary — (DOCX) [file pntd.0010697.s005.docx]

**Further detail and presentations of the scabies data within the Togo study**

**Table: lesions sites in scabies patients, in children and in adults N (%)**

|  | **Children (˂18 years)**  **(n = 55)** | **Adults (≥18 years)**  **(n = 31)** | **Total**  **(N = 86)** |
| --- | --- | --- | --- |
|  |  | | |
| **Site of lesions** |  |  |  |
| Buttocks | 46 (83.6) | 22 (71) | 68 (79.1) |
| Wrists | 36 (65.5) | 29 (93.5) | 65 (75.6) |
| Interdigital spaces | 33 (60) | 23 (74.2) | 56 (65.1) |
| Breasts | 16 (29.1) | 17 (54.8) | 33 (38.4) |
| Forearms | 20 (36.4) | 11 (35.5) | 31 (36) |
| Arms | 18 (32.7) | 8 (25.8) | 26 (30.2) |
| Thighs | 16 (29.1) | 5 (16.1) | 21 (24.4) |
| External genitalia | 11 (20) | 8 (25.8) | 19 (22.1) |
| Peri-umbilical region | 11 (20) | 6 (19.4) | 17 (19.8) |
| Feet | 12 (21.8) | 2 (6.5) | 14 (16.3) |
| Palms of the hands | 3 (5.5) | 2 (6.5) | 5 (5.8) |
| Feet plants | 3 (5.5) | 0 (0) | 3 (3.5) |
| Other | 3 (5.5) | 0 (0) | 3 (3.5) |


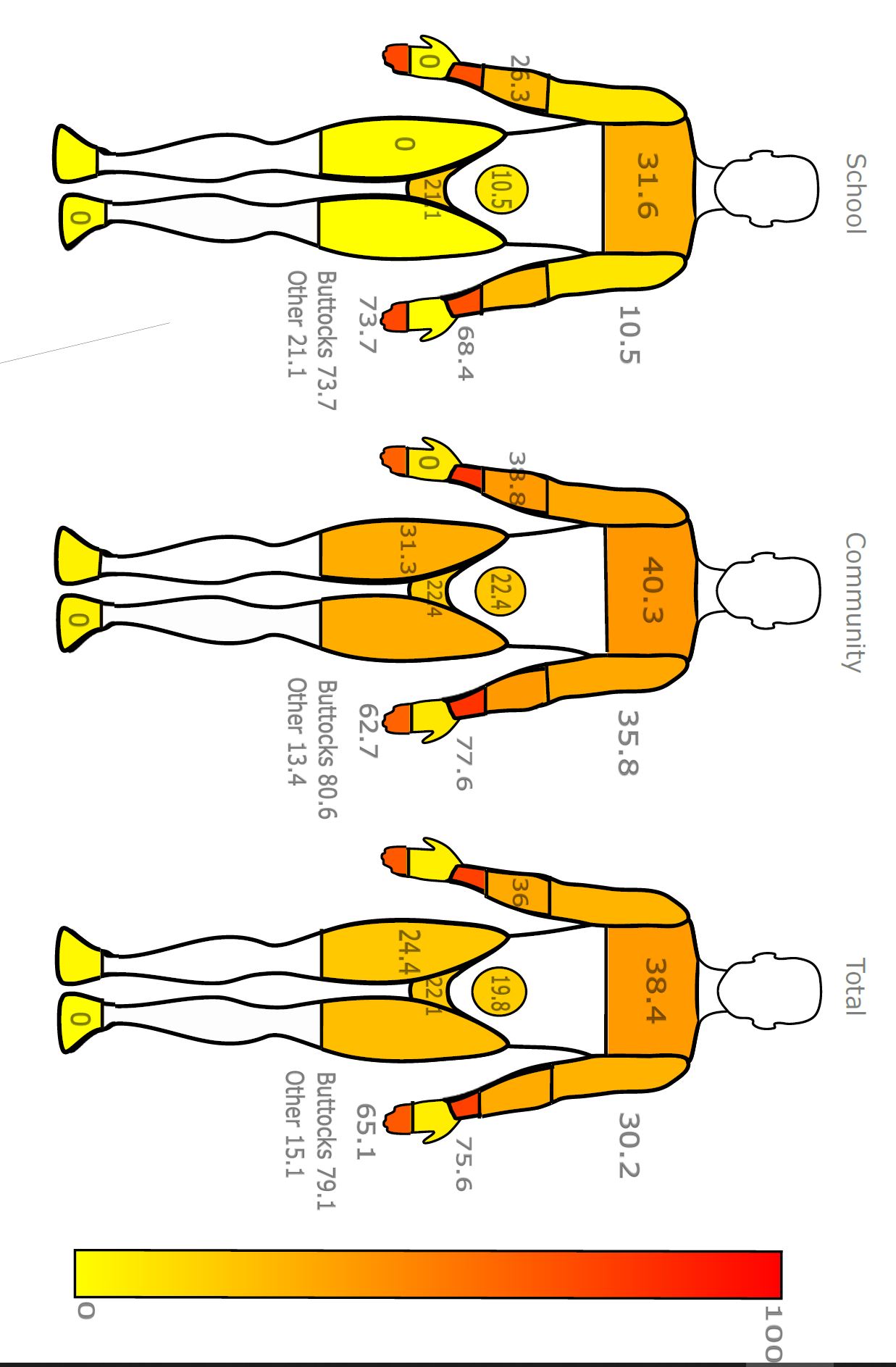


*Supplementary figure. Anatomy heat map showing clinical presentation of scabies across school and community study sites. Image drawn within our research group, the Clinical Informatics Research Unit at University of Southampton.*


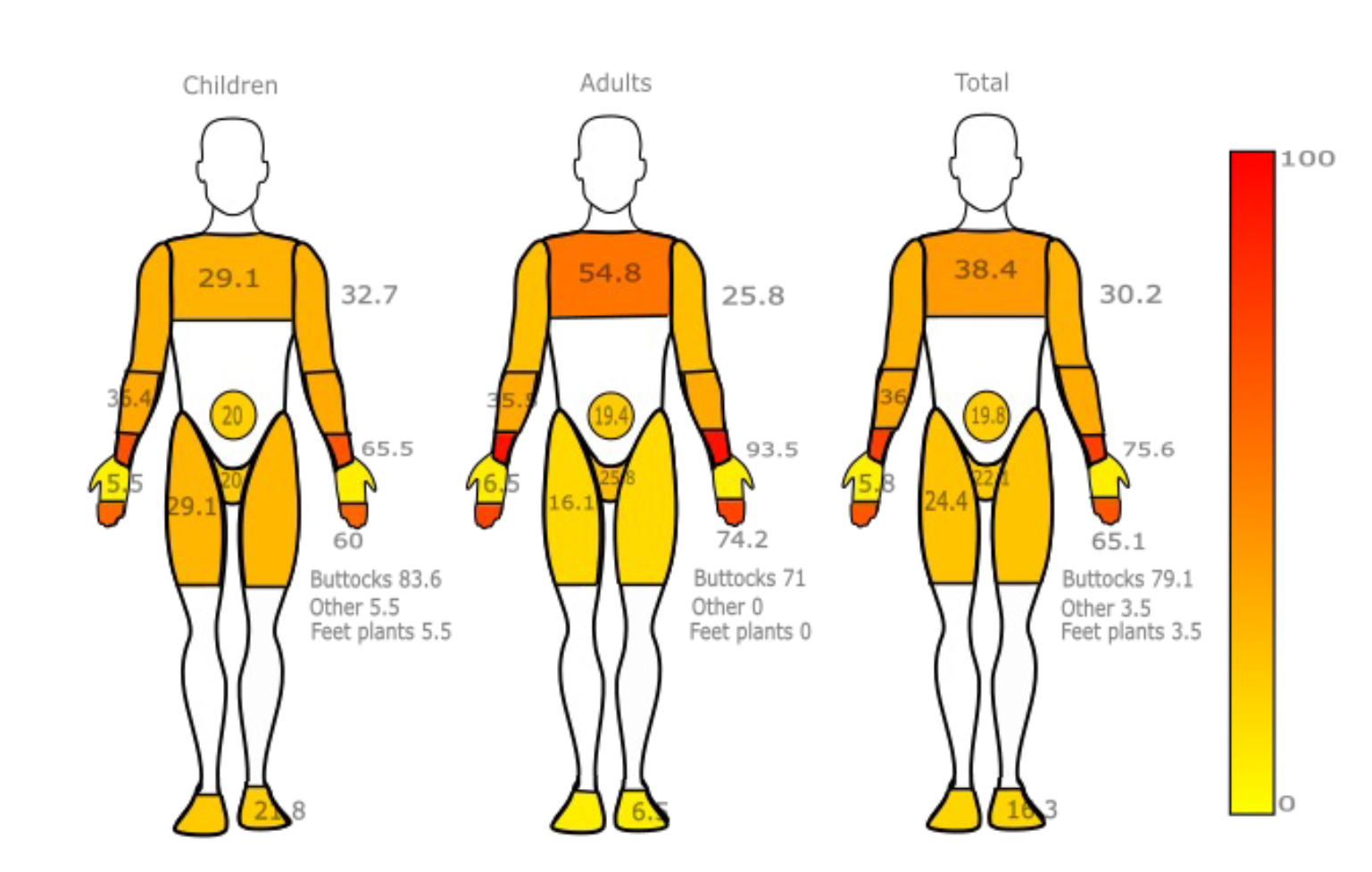


*Supplementary figure. Anatomy heat map showing clinical presentation of scabies in children and adults within our school and community study populations. Image drawn within our research group, the Clinical Informatics Research Unit at University of Southampton.*
