# Supplementary material for: Prevalence of skin Neglected Tropical Diseases and superficial fungal infections in two peri-urban schools and one rural community setting in Togo
Source: PLoS Negl Trop Dis. 2022 Dec 19;16(12):e0010697. doi: 10.1371/journal.pntd.0010697 (PMC9810153; doi:10.1371/journal.pntd.0010697)
Supplement: Attachment — Submitted filename: author response to reviewers v2.docx [file pntd.0010697.s006.docx]

**Author response to reviewers**

Many thanks to the reviewers for their very helpful corrections and suggestions. See below for our responses.

We also have now additionally translated the revised manuscript intro French, with the aim of both English and French versions being available, should this be accepted for publication.

**Addendum, 9 November – we have added further content below around editorial modifications received on 2 November. All requested modifications have been addressed.**

| **Methods** |  |
| --- | --- |
| Reviewer #1: (No Response) | N/A |
| Reviewer #2: This cannot really be called a prevalence study as almost 40% of the school children invited did not attend because of lack of parental consent and from later figures only 1/3 of the community population was investigated. It is not clear therefore whether attendees were truly respresentative of the whole population So without understanding whether those who did not attend had the same rate and distribution of skin disease and Skin NTDs we do not have a true picture of prevalence. The authors briefly discuss this at the end in relation to the school population. | We have enhanced the limitations section about participation.  We have also added in the sample size calculations into the methods (both school and community did meet the required sample size)  Given that, and given limitations are also better discussed in this revision, we disagree with the suggestion that we cannot refer to prevalence. |
| I note that medicines were provided free of charge – was this for the common skin diseases ? | These included anti-scabies and anti-fungal medicines. We have added the list into supplementary info, and added mention of this into the methods. |
| “Where necessary, individuals were referred to the appropriate service in the healthcare system” . Is this in reference to NTDs such as leprosy ? | We have added mention into the methods (line 98) “For example, leprosy patients were referred to specific leprosy care centers at the district hospital.” |
| What were the other skin infections ( briefly) ? | We presume this is referring to ‘other skin infections’ mention in the abstract. Here, we have added in reference to mycoses |
| How was the issue of stigma approached – for instance what questions were asked ? This is critical to understanding the implications of this work and more detail is needed. I presume it is this one from the supplementary material Si «oui» à la question sur la stigmatisation, le participant à l’étude n’a-t-il pas assisté à un ou plusieurs jours de travail ou d’école en raison de la stigmatisation?. There should be a comment in the text – but is this sufficient evidence to indicate stigmatisation ? | We note that we had not uploaded the English version of the data collection form too, apologies. This is now added in supplementary information.  We have also added in the follow text into the methods (line 141), with an accompanying citation  “The question on the data collection form was “If a skin infection is diagnosed, did the study participant experience stigma as a result of their infection?” (S2). The dermatologist asked if the individual was being treated differently or unfairly as a result of their skin infection, a common approach to gain an indication around levels of stigma” |
| Reviewer #3: Hypothesis is clearly stated, and the study design is appropriate to investigate the stated objective. Please clarify the "community setting" (line 22-25) in the introduction. | I think the line count numbering between reviewer and author is not the same (for us, introduction covers lines 66-85).  However, we think this is referring to this sentence “The aim of this study was to determine the prevalence of skin NTDs in two schools and one rural community setting,”  We have added “that of village of Ndjéi, in rural north-east Togo” to the text here, and there is more description about Ndjei in the methods. |
| Given that more than 50% of the population in the rural setting were school children, it would be good to add subset analysis of school children vs non school children in the rural setting, etc. | Excellent suggestion, thank you. This has now been done, and is available within supplementary information (including some new anatomy heat map vizualisations of the scabies presentations) |
| Please explain how the choice of schools and the village was random. | We have corrected this. The abstract now reads ‘Study sites were purposively selected’ |

**Results**

| Reviewer #1: (No Response) | N/A |
| --- | --- |
| Reviewer #2: Page 14 scabies furrows - ? Burrows. | Yes, thanks for the correction, we do mean burrows!  Change made. |
| This sentence is a bit confusing – “These fungal diseases are not always considered as neglected diseases, but they have previously been referred to in the WHO list of cutaneous NTDs”. Superficial fungal infections are not listed by WHO as Skin NTDS. The issue here is that if skin examination is used as an entry point for NTD detection, for instance as in the new Framework programme published this year, it is a pragmatic and practical approach to include the common prevalent skin diseases such as superficial mycoses when dispensing treatment rather than to ignore them. So they are included in, for instance, the WHO App and training manual but are not listed as NTDs | We have revised that sentence to  “The WHO list of cutaneous NTDs previously referred to ‘fungal infections’.”  Anecdotally from a few colleagues, this did cause confusion about ‘what fungal diseases should we include when referring to NTDs?’. It is now clarified much better in WHO documents such as the NTD roadmap. |
| Reviewer #3: The analysis of the data, especially the given percentages are not clear and partly faulty. Please correct. State clearly what the denominator is. Line 34 the percentages in parentheses behind the numbers are incorrect instead of 6.7% it should for example be 4.4% | We have double-checked the numbers, and make some minor revisions e.g. table 2.  We have also revised and provided denominator in the abstract, and rephrased the sentence to make it clearer . The new sentence is  “Within the school setting, there were 20 individuals with NTDs (4.4% of 447 participants), and 85 NTDs (8.9%) from 954 community participants.” |
| In the text it is described, that 14 patients had more than 1 skin infection, this is not reflected in table 1 and table 2 the number of skin infections plus the number of patients without a skin infections adds up to 100%, if there were 14 patients with more than 1 skin infection, there would need to be at least 14 skin infections more, or the number of patients without a skin infection would need to be higher. | We have corrected this to “14 participants had more than one skin infection at the same time” (rather than concurrent NTDs) |
| Table 3 last row, the percentages don't fit. Please recheck all percentages and make clear the percentage of what (sub)population it is | The numbers are correct, but the way we presented them is confusing!  The proportions are in relation to the row immediately above.  We have revised the table structure to make this clearer |

**Conclusions**

| Reviewer #1: (No Response) | N/A |
| --- | --- |
| Reviewer #2: In discussing the accuracy of the diagnosis of yaws can you provide an example of a disease that might be confused with yaws ? | We have added a citation into the discussion (line 241, page 18), with this sentence  “The skin presentation of primary yaws can be similar to cutaneous leishmaniasis or mycobacterial disease.” |
| The authors point out the main limitation of this study which is the comparatively low participation rate in schools . Without examining non attenders it is not possible to comment on the accuracy of the prevalence figures although these may not have been affected. But it would be helpful to explore and suggest ways of increasing participation for the future as this is critical information for the success of Skin NTD programmes. | We briefly expand upon existing content in the limitations, for example with this -  “With school and community health programs such as mass drug administrations or vaccine rollouts, uptake is typically higher where there has been extensive community engagement.^31^ A sustained programme of health promotion, across school and community settings, may encourage a proactive approach to healthcare-seeking behaviour, plus participation in research studies.” |
| Reviewer #3: Please add in the discussion a part discussing other possibilities/more in depth how the low percentage of school children could have been effected by the fact, that people with skin NTD rather stay at home, among other theories maybe. | See also responses to other reviewers, but we have added content into the limitations section, including this sentence “If children with a skin infection are indeed being stigmatized, then they may have been more likely to be absent at the time of our study, and thus prevalence may be underestimated” |
| Add, that in the rural setting a subset was investigated, because these were people, who were already coming to the health center, this is a subset of the population, which could differ from the overall population. | Reference to this has also been added into the limitations section |
| Line 258 - add percentage of people reported stigma/all people with NTDs | Apologies, as per mentioned previously, the line numbering system for reviewers does not appear to match the line numbers for our manuscript.  However, we have revised mention of stigma numbers in the table, and improved presentation to clarify percentages. |
| Line 287 - please reiterate further on the limitations of the study, e.g. on the small sample size | Limitations section has been enhanced, including (here) with this sentence  “The sample analysed here may not be representative of these populations as a whole.” |

**Editorial and Data Presentation Modifications?**

| Reviewer #1: (No Response) | N/A |
| --- | --- |
| Reviewer #2: (No Response) | N/A |
| Reviewer #3: Consider using a map, which shows also other cities in Togo, to show where the remote community (Ndjéi) is. | We do already have a map (figure 1) showing Ndjei, and Lomé (Alongside the location of Togo itself)  We have added into the methods mention that “The nearest large town is Atakpamé, approximately 160km away.” |
| Line 69 - add how climate and rainfall are risk factors and what climate is a risk factor. | We have revised ‘climate and rainfall’ to humidity (with the accompanying reference). |

**Summary and General Comments**

| Reviewer #1: Line 77 - not all of the skin NTDs are communicable (for example BU is likely from the environment as are most of the fungal diseases) and it is not clear that it is the absence of dermatologists that creates a barrier to their control. | Good point! We have revised this to ‘mostly communicable’.  We have removed mention of control from ‘diagnosis and control’. |
| --- | --- |
| Line 79- the reference appears to be about leprosy; I am not aware of any good evidence that increased access to dermatologists (which is undoubtedly a good thing) helps break chains of transmission of yaws or scabies. Please reword. | We do not suggest that increased dermatologists break chain of transmission (mention of dermatologists was the previous sentence and citation).  However, we agree that it can be better worded the full sentence is “Educational interventions are known to facilitate early detection of stigmatising or contagious diseases such as leprosy, and to prevent their spread in the community” |
| Methods - were dermatologists of both genders available? | We have added in this sentence into the methods  “Both male and female dermatologists were available across school and community settings.” |
| How was the random selection of households done? Simple random sampling? Random walk? Where was the randomisation list generated from. | We have added the following into the methods – “Household selection was via the random walk method, with guidance from local health service colleagues (for example whether to purposively exclude a household).” |
| It is not possible to make a clinical diagnosis of yaws reliably - this has been shown in numerous papers. Similarly BU can not be diagnosed reliably solely on clinical grounds. I would suggest you reword the methods to make clear that a suspected diagnosis could be made but not an actual one. Tables and references to these diagnoses in the results and discussion also need to be amended. - In addition for yaws it is critical to differentiate between papillomas (specific, likely to be yaws) and ulcers (non specific, most commonly wont be yaws) | We have added mention of ‘suspected’ into methods and discussion (including limitations), plus also amended e.g. abstract. |
| How were the questions on stigma asked/collected? Is this a validated tool. | As per other response, we have added the English-language data collection form into supplementary, and clarified the question asked (which is based upon existing literature, and indeed also a question we asked to gain insight into stigma in a separate long covid study in Ghana) |
| The information on consent is provided twice in duplicate (line 101 and then again in the ethics section) please remove one of these. | Thank you, we have removed the mention from within the ‘school environment’ paragraph, leaving the content within the ethics section |
| Results - what is a furrow - I presume this is a typo and is meant to be burrow. | Yes, thanks for the correction, we do mean burrows!  Change made. |
| Discussion - I do not think you should be referring to a diagnosis of yaws here and certianly not claiming it was common without a) some details on papillomas vs ulcers and b) given the complete absence of diagnostic testing | As per other response, we have revised mention of yaws to ‘suspected’. |
| Line 225 I am not aware that WHO has ever regarded superficial mycoses as NTDs and the reference you provide for this statmeent (13) does not suggest this either. Please remove/reword this. | As per other response, we have clarified the sentence, albeit the WHO (And the reference we provided) did specifically make mention to ‘fungal infections’ without then specifying it solely referred to e.g. deep-tissue mycoses. Anecdotally, this has caused confusion among many healthcare workers and policymakers in the LMIC setting who focused on NTDs.  The WHO are now much clearer in their definition. |
| The discussion is quite long (>1200 words) and I would suggest benefit from editing to make it more concise. | The discussion has been revised (both additions and removal/editing of text). However, if editorial input suggests that further content should/could be removed, then we will do so. |
| Where are data available from - please provide this information | The dataset has now been uploaded to <https://doi.org/10.6084/m9.figshare.21276693.v1> with statement added to the end of the manuscript. |
| Reviewer #2: While I recognise the difficulty of carrying out studies of this nature some attempt should be made to show that the population examined were representative of the whole | We have now added in mentions of sample size into the methods (We did meet sample size requirements in both school and community).  There is further discussion around study limitations, and the ‘unknowns’ around those who did not participate (an important point, of course) |
| Reviewer #3: Good study overall, which gives insight into an understudied topic and adds value to the community, adding important information regarding the prevalence of skin NTDs in another african country. | Many thanks for the feedback. |
| The study is limited to a low number of participants and the methods used are subjective. Not sufficient information about the training of the doctors is given, and as clinical diagnoses were the only method to diagnose a high false positive/negative rate must be assumed. | We have added ‘suspected’ to diagnoses of yaws and Buruli ulcer. As per other responses, the limitations section is greatly enhanced in this revision.  We do already mention in the methods that ‘Each of the five dermatologists had a minimum of five years of experience in dermatological medical practice. ‘ |
| The relevance of the sub-analysis is questionable as no major conclusions were found. | We are unsure as to which part of the analysis is being referred to here.  If table 2, school v community, then one reviewer has indeed suggested an additional similar sub-analysis. If table 3, scabies presentations, then it is the NTD we have most data on, and we make reference to this in the discussion.  If editorial feedback is that some content should be removed altogether, or to the appendix, we will do so. |
| Research and publication ethics seem to have been respected. A good study requiring some more work on the analysis part. | Many thanks for the feedback. |
| Data for the socio-demographic information collected lacks. | Looks like the end of this comment is missing? No action here from us, but please let us know if we need to edit/correct anything further! |

**Editorial feedback/comments**

| Figure Files: While revising your submission, please upload your figure files to the Preflight Analysis and Conversion Engine (PACE) digital diagnostic tool, https://pacev2.apexcovantage.com. PACE helps ensure that figures meet PLOS requirements. To use PACE, you must first register as a user. Then, login and navigate to the UPLOAD tab, where you will find detailed instructions on how to use the tool. If you encounter any issues or have any questions when using PACE, please email us at figures@plos.org. | We have done so, and the image file from PACE is now attached to this revised submission |
| --- | --- |
| Data Requirements: Please note that, as a condition of publication, PLOS' data policy requires that you make available all data used to draw the conclusions outlined in your manuscript. Data must be deposited in an appropriate repository, included within the body of the manuscript, or uploaded as supporting information. This includes all numerical values that were used to generate graphs, histograms etc.. For an example see here: <http://www.plosbiology.org/article/info%3Adoi%2F10.1371%2Fjournal.pbio.1001908#s5>. | We have uploaded the data, and made mention of the location/DOI number at the end of the manuscript |
| Reproducibility: To enhance the reproducibility of your results, we recommend that you deposit your laboratory protocols in protocols.io, where a protocol can be assigned its own identifier (DOI) such that it can be cited independently in the future. Additionally, PLOS ONE offers an option to publish peer-reviewed clinical study protocols. Read more information on sharing protocols at <https://plos.org/protocols?utm_medium=editorial-email&utm_source=authorletters&utm_campaign=protocols> | N/A |

**Further editorial modifications received as of 2 November**

| 1) Please upload all main figures as separate Figure files in .tif or .eps format. | This has been done |
| --- | --- |
| 2) We note that your "Supporting Information (S2)" files are duplicated on your submission. Please remove any unnecessary or old files from your revision, and make sure that only those relevant to the current version of the manuscript are included. | This has also been done |
| 3) Please ensure that all author names are spelled correctly in the manuscript file, including the use of a middle name/initials, and any special characters (accents, umlauts, etc.). Please place first/given names before last/family names. All authors must have a first and last name. We noticed that " Michael G Head,Kafouyema Katsou" are spelled differently between the manuscript file and submission form. Please be aware that correct spelling and formatting of author names in the article file is vital for accurate indexing in PubMed. | The names on the tracked changes, clean copy, French version and the online submission system now match, |
| 4) We do not publish any copyright or trademark symbols that usually accompany proprietary names, eg (R), (C), or TM (e.g. next to drug or reagent names). Please remove all instances of trademark/copyright symbols throughout the text, including R© on line no 157. | This has been done |
| 5) Thank you for stating " An anonymized dataset can be downloaded from <https://doi.org/10.6084/m9.figshare.21276693.v1>. More information is also available from the study team, via the correspondence author".  In the online submission form you indicate that your data is available from a co-author on this manuscript. According to our Data Policy, data must either be included with the submission, be placed in a public repository, or available from a third party contact point who must not be an author on the manuscript. Please revise your data statement to a non-author institutional point of contact, such as a data access or ethics committee, and send this to us via return email. Please also include contact information for the third party organization (this contact should be a central address and not a named person), and please include the full citation of where the data can be found. | The data availability statement now matches across tracked changes, clean copy, and French manuscripts. It refers specifically to the DOI link where the anonymised dataset can be found. |
| 6) Some material included in your submission may be copyrighted. According to PLOS’s copyright policy, authors who use figures or other material (e.g., graphics, clipart, maps) from another author or copyright holder must demonstrate or obtain permission to publish this material under the Creative Commons Attribution 4.0 International (CC BY 4.0) License used by PLOS journals. Please closely review the details of PLOS’s copyright requirements here: PLOS Licenses and Copyright. If you need to request permissions from a copyright holder, you may use PLOS's Copyright Content Permission form.  Please respond directly to this email and provide any known details concerning your material's license terms and permissions required for reuse, even if you have not yet obtained copyright permissions or are unsure of your material's copyright compatibility. Once you have responded and addressed all other outstanding technical requirements, you may resubmit your manuscript within Editorial Manager. | Email sent confirming no copyright issues on 9 November 2022. |
| 1) Thank you for providing the following information [For Map]: "This is an image drawn within our research group, the Clinical Informatics Research Unit at University of Southampton". Please add this information to the Figure1 legend and also to the S1 French language version of this manuscript (Figure 1) Legend | This has been done, across all manuscripts |
| 2) [S5 presentation of scabies data]: Please confirm whether you drew the images / clip-art within the figure panels by hand. If you did not draw the images, please provide (a) a link to the source of the images or icons and their license / terms of use; or (b) written permission from the copyright holder to publish the images or icons under our CC BY 4.0 license. Alternatively, you may replace the images with open source alternatives. | To confirm these images were drawn within our research unit. We have added statements to the legends in the supplementary file. |

 
